# The Necessity of Stool Examination in Asymptomatic Carriers as a Strategic Measure to Control Further Spread of SARS-CoV-2

**DOI:** 10.3389/fpubh.2020.553589

**Published:** 2020-10-30

**Authors:** Hamed Mirjalali, Ehsan Nazemalhosseini-Mojarad, Abbas Yadegar, Seyed Reza Mohebbi, Kaveh Baghaei, Shabnam Shahrokh, Hamid Asadzadeh Aghdaei, Mohammad Reza Zali

**Affiliations:** ^1^Foodborne and Waterborne Diseases Research Center, Research Institute for Gastroenterology and Liver Diseases, Shahid Beheshti University of Medical Sciences, Tehran, Iran; ^2^Gastroenterology and Liver Diseases Research Center, Research Institute for Gastroenterology and Liver Diseases, Shahid Beheshti University of Medical Sciences, Tehran, Iran; ^3^Basic and Molecular Epidemiology of Gastrointestinal Disorders Research Center, Research Institute for Gastroenterology and Liver Diseases, Shahid Beheshti University of Medical Sciences, Tehran, Iran

**Keywords:** COVID-19, stool examination, children, asymptomatic carriers, public health

In early December 2019, a novel enveloped RNA beta coronavirus named severe acute respiratory syndrome coronavirus 2 (SARS-CoV-2) was identified in Wuhan, and has rapidly spread on a global scale. Soon after, the novel coronavirus was designated the name COVID-19. Finally, the World Health Organization (WHO) announced the outbreak as a pandemic on March 11. Although the prevalence rate of asymptomatic subjects appears to be up to 45% (1), the prevalence of symptomatic infection in children younger than 10 years is reported to be zero (2, 3). The most common symptoms of COVID-19 are fever, dry cough, and dyspnea (4). However, gastrointestinal symptoms like diarrhea, nausea, vomiting, and abdominal discomfort are also being reported in <30% of patients (5, 6). A body of evidence shows that binding of the virus to the host cell receptors may play a key role in the pathogenesis of infection. Actually, SARS-CoV-2 is required to bind to the angiotensin-converting enzyme 2 (ACE2) to enter the cells (7).

ACE2 is a type 1 integral membrane glycoprotein that is expressed in almost all tissues. The highest expression of ACE2 is observed in the lungs, arteries, heart, kidneys (8), and also in enterocytes throughout the ileum and colon (9) which may increase the probability of replication of the virus in the intestine, shedding in stool, and the further distribution of SARS-CoV-2 in the environment (Figure 1). During the COVID-19 pandemic, a couple of studies reported on the presence of viral RNA in stool samples. Accordingly, the prevalence of SARS-CoV-2-positive stool in COVID-19 patients varies from 36 to 53% (10). Xiao et al. (11) analyzed stool samples of 73 SARS-CoV-2-infected patients and showed that more than half of them were positive for SARS-CoV-2 RNA. Unexpectedly, 23.29% of patients were positive for SARS-CoV-2 RNA while their respiratory samples were negative (11). Holshue et al. (12) reported the first case of COVID-19 in the US in a 35-year-old man who suffered from abdominal discomfort on his second day of hospitalization and his stool sample was positive for the virus using a real-time reverse-transcriptase polymerase chain reaction (rRT-PCR) test, and remained positive until day 7 (the stool samples on day 11 and 12 were not tested). The presence of SARS-CoV-2 RNA in stool samples was also confirmed in a couple of studies in symptomatic and even asymptomatic carriers (13–16). However, the virus may shed from stool samples for days, even after clinical symptoms have disappeared and patients have been discharged from hospital or quarantine (16, 17). Surprisingly, Lamers et al. (18) demonstrated the growth and replication of SARS-CoV-2 in human small intestine organoids (hSIOs) which provides evidence for the role of stool in the distribution of SARS-CoV-2. Therefore, although the fecal-oral transmission of SARS-CoV-2 has not been proven, a couple of studies have highlighted concerns about shedding and the distribution of active virus particles through the stool of patients, particularly asymptomatic subjects (9, 10, 18). Nevertheless, it seems that there is no correlation between gastrointestinal symptoms and the positive rate of stool tests (10).

**Figure 1:**
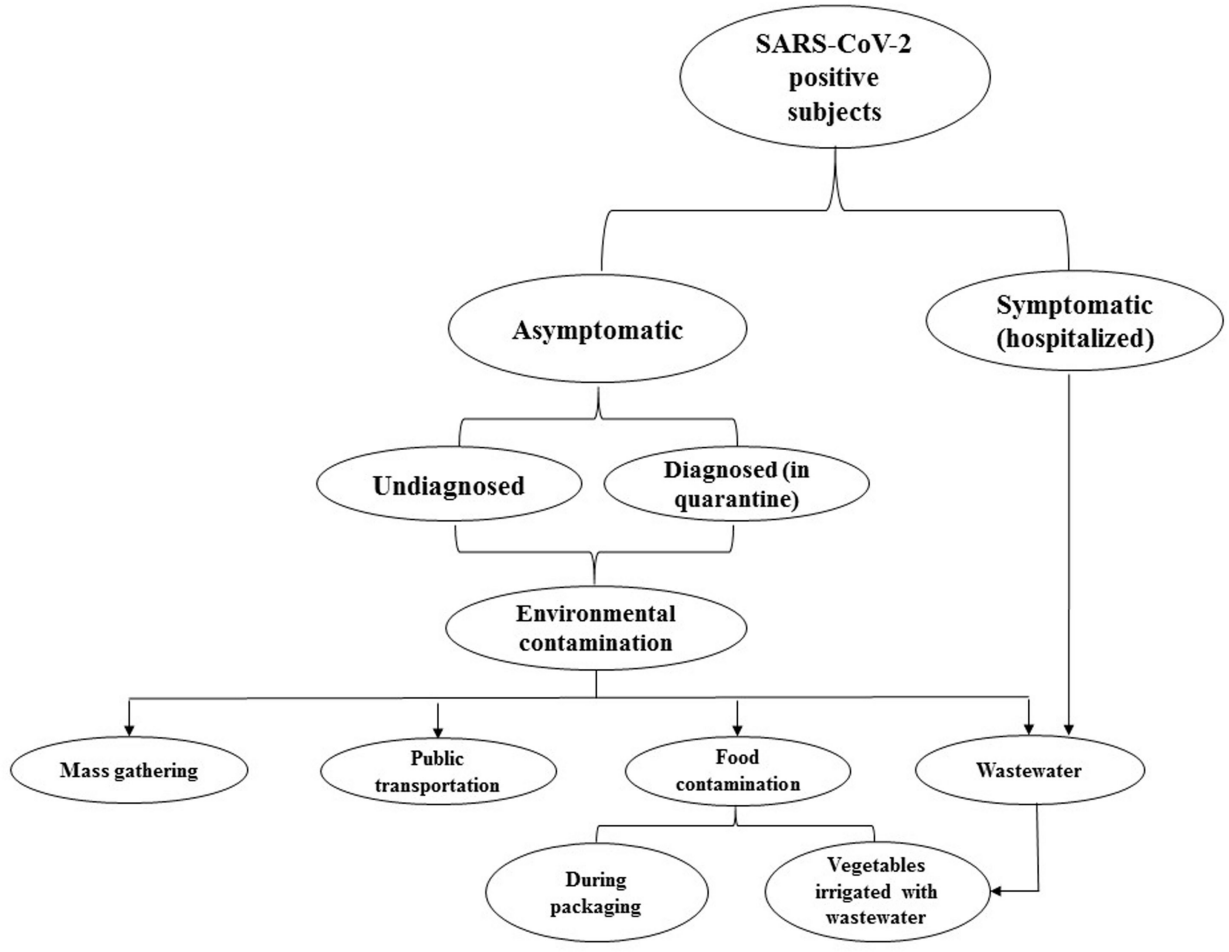
This flowchart represents the probable environmental distribution of COVID-19. Accordingly, asymptomatic subjects, particularly those who are undiagnosed, can disperse infective virus particles. Discharging the virus from stool may increase the load of SARS-CoV-2 in wastewater. Therefore, concerning the insufficiency of current wastewater treatment systems in eliminating the virus, wastewater can lead to the further distribution of SARS-CoV-2 in the environment.

From the public health point of view, a large percentage of the infected subjects do not represent any clinical symptoms but may still shed the infecting virus particles from their stool samples (16, 17). In this regard, cross-contamination may happen in general laboratories that routinely investigate stool samples for microbial agents other than SARS-CoV-2. Therefore, the laboratory technicians who work on stool samples could be an at risk group for the disease. Additionally, another emerging source of the infection might be kindergartens where children under 6 years old regularly visit. Notably, it seems that most of the children do not have symptoms similar to those that are frequently reported in adults (19) which highlights the importance of children as asymptomatic carriers. It was reported that this group of infected subjects may discharge the virus particles in stool for an extended amount time even though their throat swabs test negative (16, 17). Therefore, these asymptomatic carriers may be an important part of the transmission chain. In another words, teachers and other workers in kindergartens, who are in close contact with the infected children, might be infected with COVID-19 while at work and transmit the infection to their families or communities (Figure 2).

**Figure 2:**
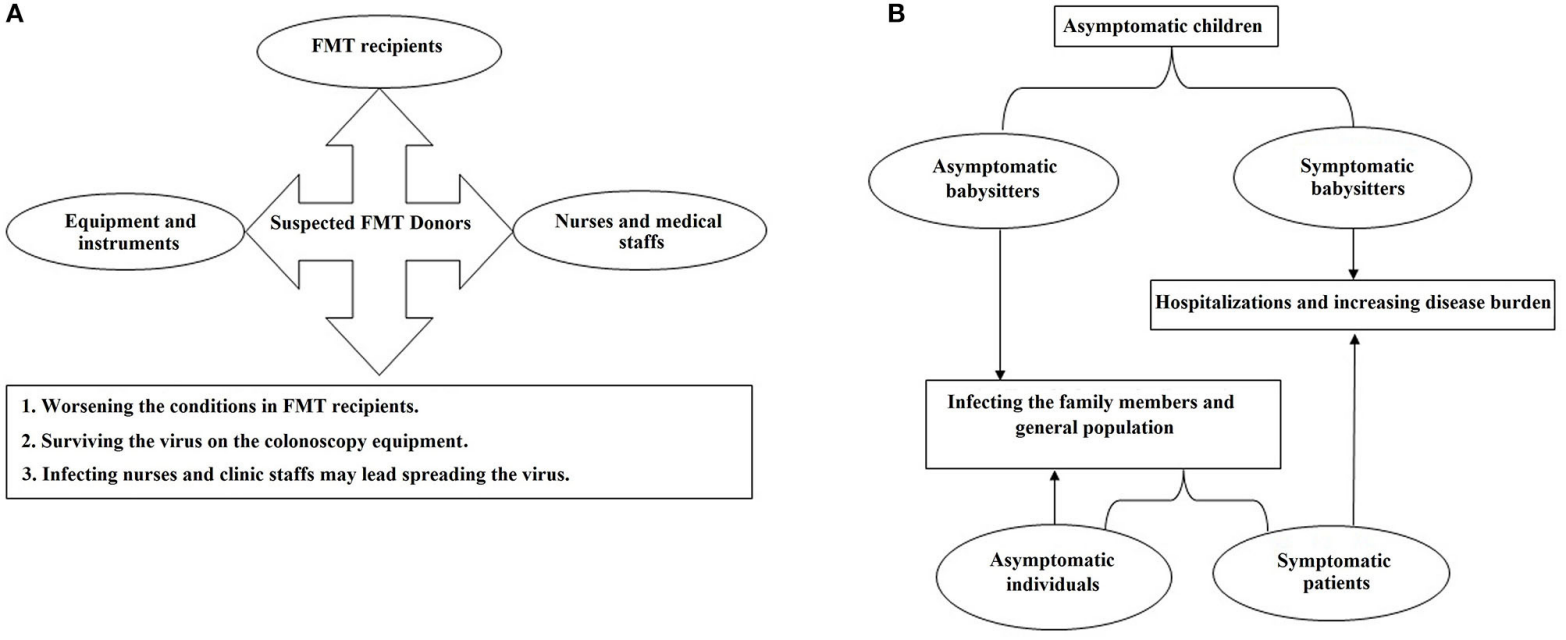
The flowcharts of the probable distribution scenarios for COVID-19 which may happen in **(A)** gastroenterology clinics and **(B)** primary schools and kindergartens.

Moreover, during fecal microbiota transplantation (FMT), the presence of COVID-19 may increase the risk of fecal transmission to FMT recipients (20, 21) or operators. Furthermore, colonoscopy instruments could be contaminated with the virus and remain infective during the next procedure (Figure 2). Employees of stool banks who work on the processing and storage of the stool samples taken from COVID-19-asymptomatic donors might be another at-risk group.

Therefore, we believe that stool examination for COVID-19 should be considered as a screening strategy during the pandemic and also in the post-COVID-19 era, particularly in kindergartens, primary schools, gastroenterology clinics, stool banks, and general laboratories to prevent the further spread of the virus.

## Author Contributions

HM, EN-M, AY, SRM, KB, SS, HAA, and MRZ: conceptualization and writing. All authors contributed to the article and approved the submitted version.

## Conflict of Interest

The authors declare that the research was conducted in the absence of any commercial or financial relationships that could be construed as a potential conflict of interest.
